# Rural–Urban and Demographic Inequalities in Dental Pathology and Diagnostic Patterns in Romanian Emergency Care: A Retrospective Tertiary Clinical Cohort Study

**DOI:** 10.3390/healthcare14131914

**Published:** 2026-07-01

**Authors:** Daniela Roxana Vintilă, Tudor Olariu, George Andrei Drăghici, Gabriela-Victoria Mnerie, Iustin Olariu

**Affiliations:** 1 Department of Otorhinolaryngology, Faculty of Medicine, “Victor Babes” University of Medicine and Pharmacy, Eftimie Murgu Square 2, 300041 Timisoara, Romania; vintila.daniela@umft.ro; 2 Faculty of Pharmacy, Eftimie Murgu Square 2, 300041 Timisoara, Romania; olariu.t@umft.ro; 3 Research Center for Pharmaco-Toxicological Evaluations, Faculty of Pharmacy, “Victor Babes” University of Medicine and Pharmacy, Eftimie Murgu Square No. 2, 300041 Timisoara, Romania; 4 The National Institute of Research—Development for Machines and Installations Designed for Agriculture and Food Industry (INMA), Bulevardul Ion Ionescu de la Brad 6, 077190 București, Romania; gabriela.mnerie@islavici.ro; 5 Department of Computers and Exact Sciences, Faculty of Engineering, “Ioan Slavici” University of Timișoara, 144 Dr. Aurel Păunescu-Podeanu Street, 300587 Timisoara, Romania; 6 Department of Dentistry, Faculty of Dentistry, “Vasile Goldiş” Western University of Arad, 94–96 Revoluţiei Blvd., 310025 Arad, Romania; olariu.iustin@uvvg.ro

**Keywords:** oral health, endodontics, periodontal diseases, dental caries, tooth diseases, emergency medical services

## Abstract

**Background**/**Objectives**: This study investigated the independent and interactive effects of age, sex, and area of residence on dental diagnostic and anatomic patterns among emergency dental patients. **Methods**: Clinical data were collected from 1650 patients treated at the Arad County Emergency Clinical Hospital and analyzed using multivariate logistic regression. Dental diagnoses were grouped into carious lesions, endodontic conditions, periodontal diseases, acute infections, and other dental pathologies. Affected teeth were classified as anterior, premolar, or molar. **Results**: Middle-aged patients (40–59 years) showed lower odds of dental caries (OR = 0.21, *p* = 0.009) and endodontic conditions (OR = 0.42, *p* = 0.011), but higher odds of periodontal diseases (OR = 2.02, *p* < 0.001) compared to young adults (<40 years). Older age (≥60 years) was associated with reduced risk of endodontic emergencies (OR = 0.58, *p* = 0.021) but higher risk of periodontal diseases (OR = 1.92, *p* = 0.026). Rural residence was associated with higher odds of endodontic (OR = 1.57, *p* = 0.048) and periodontal pathology (OR = 1.76, *p* = 0.045). The endodontic effect differed by sex, with rural men showing higher odds of this outcome versus urban women (OR = 2.03, *p* = 0.033). Male patients were more likely to have affected anterior teeth (OR = 1.62, *p* = 0.045), whereas their molars were less frequently affected (OR = 0.69, *p* = 0.042). **Conclusions**: Age and rural residence were significantly associated with variations in advanced dental pathology patterns within this emergency cohort. Sex modified the association between area of residence and endodontic conditions and was independently associated with patterns of tooth involvement. Our findings identify rural men from this cohort as a high-risk subgroup and emphasize the need for targeted public health programs in rural communities.

## 1. Introduction

With an estimated global prevalence affecting nearly 3.5 billion individuals, dental diseases exert an annual macroeconomic burden exceeding 700 billion US dollars [1,2]. Untreated caries, periodontal diseases, and endodontic conditions are the dominant contributors to dental morbidity, often occurring as sequential or synergistic stages of oral disease progression [3,4,5]. Dental caries typically represent the earliest pathological insult affecting the dental apparatus and frequently initiate downstream endodontic pathology and eventual tooth loss [1,6,7]. Endodontic conditions involve microbial invasion and colonization of the pulp chamber and reflect progression into irreversible tooth pathology and advanced internal tissue compromise [7,8]. Periodontal diseases damage the tooth-supporting structures, leading to tooth mobility, structural deterioration, and tooth loss [9,10,11,12]. Despite recent advances in preventive dentistry, these manageable conditions still constitute the majority of cases attending the emergency dental service.

Emergency services offer valuable real-world insight into advanced dental pathology since most patients present with acute symptoms or complications of late-stage conditions [13,14]. Delayed healthcare-seeking behavior and structural barriers to preventive services are among the major drivers of dental emergency service utilization. In fact, this metric is considered an indirect indicator of failure in preventive care systems [15,16]. Multiple sociodemographic factors influence dental care utilization patterns [9,10,11,12]. Age functions as a surrogate marker of cumulative dental risk burden leading to complex dental emergencies rather than simply representing a chronological index [13]. Sex differences also shape these patterns, with females showing higher preventive dental care utilization and males more frequently postponing care [14]. On the other hand, urban residents generally benefit from greater provider availability, reduced unmet treatment needs, and lower dependence on emergency dental services compared to rural populations [9,10,12,15,16,17]. These rural-urban disparities may be particularly pronounced in Eastern European countries. For example, there is a substantial disparity in the distribution of dentists in Romania—2023 data show that 18,905 dentists were working in urban areas compared to only 2295 in rural areas [18], with approximately 87.6% of dental resources concentrated in urban regions [19]. It is therefore no coincidence that Romania continues to report the highest levels of unmet dental care needs in the European Union, particularly among socioeconomically disadvantaged and rural populations [20,21]. These observations are consistent with a broader burden of oral disease in Romania, where untreated caries, tooth loss, and other preventable dental conditions remain important public health concerns. Although comprehensive national data regarding oral health literacy and decayed, missing, and filled teeth (DMFT) index are limited, the high prevalence of unmet dental care needs suggests persistent challenges related to prevention, early diagnosis, and timely utilization of dental services. Furthermore, evidence from this country and the broader Eastern European region remains scarce, especially regarding tertiary emergency cohorts. Moreover, dental literature typically describes patient profiles and diagnoses, but rarely addresses more nuanced questions—for example, how patients’ area of residence, sex, age, and care pathway interact, or how these interactions relate to the type of dental emergency.

At the dental level, the picture is dual. For emergencies dominated by caries, pulp and periapical problems, posterior molars are the most affected. In contrast, traumatic injuries most commonly affect the maxillary central incisors, whereas pericoronitis predominantly involves third molars [22,23,24]. With respect to sex-based differences, available evidence is limited, fragmented, and indirect, but supports a higher prevalence of anterior teeth involvement in males and molar involvement in females [25,26,27,28,29]. Nevertheless, few studies have examined whether demographic and geographic disparities extend beyond diagnostic categories to influence tooth-type involvement.

Our main hypothesis was that age, sex, and area of residence independently and interactively shape diagnostic and anatomical patterns of advanced dental pathology in patients attending emergency dental services. We expected rural residence to increase the likelihood of dental conditions relative to urban residence and sex to modulate this association. We also anticipated a shift in pathology type with advancing age, alongside sex-based differences in the most frequently affected tooth type. To test these hypotheses, we collected data on age, sex, area of residence, dental condition, and affected tooth type in a large cohort presenting for emergency dental services at the Arad County Emergency Clinical Hospital. We integrated these data into logistic regression models, with age, sex, and area of residence serving as predictors, and dental condition and affected teeth as outcomes. Addressing these questions may help to identify high-risk population groups within this region and guide targeted local public health strategies in a setting characterized by significant unmet dental care needs.

## 2. Materials and Methods

### 2.1. Ethical Considerations

The protocol of the present study was approved by the Ethics Committee of the Arad County Emergency Clinical Hospital (IRB No. 123/2 March 2026). The study was conducted in accordance with the ethical principles outlined in the World Medical Association Declaration of Helsinki (2008 revision) and its subsequent amendments [30]. The present investigation consisted of a retrospective analysis of routinely collected clinical records. At the time of hospital admission, all adult patients (≥18 years) signed the institution’s standard consent form permitting the anonymized use of clinical information for research purposes. The presence of this consent was verified before inclusion of records in the study. The Ethics Committee subsequently approved the retrospective analysis of these data. No additional interventions, examinations, or patient contact were undertaken for research purposes. All analyses were performed using previously collected anonymized clinical records.

### 2.2. Study Design and Participants

This observational study was conducted at the Arad County Emergency Clinical Hospital—the largest tertiary care facility in Arad County, Romania. With over 1322 beds, it is also one of the largest hospitals in the country and encompasses the majority of medical specialties, including dental services. Urgent dental cases are managed on a continuous basis, with no appointment required and continuous availability of dental staff [31]. Clinical data from patients seeking emergency dental care between 01 January and 28 August 2025 were retrospectively extracted from hospital records. No prospective recruitment or study-specific data collection was performed. Of the 1760 records initially retrieved from the emergency dental database, we excluded patients younger than 18 years (*n* = 44), as well as records with missing or invalid diagnostic codes (*n* = 17), missing demographic information (*n* = 29), and duplicate data (*n* = 20). As a result, 1650 cases were included in the final analysis. The main inclusion and exclusion criteria are presented in Table 1.

Patients were categorized into three age groups: (i) young adults (<40 years); (ii) middle-aged adults (40–59 years); and (iii) older adults (≥60 years). These categories were selected to represent clinically relevant life stages and to facilitate the evaluation of age-related patterns in dental emergencies while ensuring adequate numbers of observations within each group for statistical analysis. Previous studies have demonstrated that the prevalence and clinical presentation of oral diseases vary across adulthood, reflecting the cumulative effects of aging, long-term exposure to risk factors, healthcare utilization patterns, and general health status [32,33,34].

Dental pathologies were stratified into five groups: (i) carious lesions (tooth decay), including simple caries (incipient to early cavitated lesions), deep caries (dentin involvement to extensive cavitation), and residual root/crown caries; (ii) endodontic conditions, including pulpitis, pulp necrosis, periapical abscesses, and previously treated endodontic lesions; (iii) periodontal diseases, including gingivitis, periodontitis, and clinically significant tooth mobility; (iv) acute infections, including dental abscesses, cellulitis, and other acute odontogenic infections; and (v) other dental pathologies, including edentulism, dental trauma, and non-carious lesions. This classification system was selected to reduce heterogeneity associated with individual ICD-10 codes, capture clinically relevant pathological domains, preserve epidemiological interpretability, and enhance statistical power. Similar approaches are commonly used in dental literature when the primary objective is to examine sociodemographic disparities and anatomical patterns in dental disease burden [35,36]. The K08.x diagnoses were grouped into the category “other dental pathologies” because they represent heterogeneous conditions related to tooth loss, residual dental structures, and disorders of the teeth and supporting tissues that do not fit within the primary carious, endodontic, periodontal, or acute infectious categories. Grouping these diagnoses reduced fragmentation across numerous low-frequency ICD-10 subcodes while preserving clinically meaningful distinctions and ensuring adequate sample sizes for statistical analysis.

Because the unit of analysis was the patient encounter rather than the individual tooth or lesion, each participant was assigned a single primary diagnosis. This approach avoided duplication of observations across diagnostic categories and ensured that each patient contributed only once to the regression analyses. The assigned diagnosis reflected the principal condition responsible for the emergency presentation and clinical management during that visit. In cases with multiple diagnoses, a hierarchical approach was applied. We prioritized acute infectious conditions when present, followed by endodontic pathology, periodontal disease, carious lesions, and other dental conditions. This diagnostic classification framework captures the transition from carious lesions to endodontic and infectious conditions, as well as the chronic nature of periodontal disease. Tooth types were grouped into anterior (incisors and canines), premolars, and molars to capture clinically relevant differences in disease patterns while ensuring adequate subgroup sizes for statistical analysis.

### 2.3. Statistical Analysis

We first applied separate logistic regression models per each diagnostic category, using age, sex, and area of residence as predictors. This approach aimed to improve interpretability and facilitate subgroup interaction analyses, allowing us to understand distinct pathology-specific disparities. Sex and area of residence were entered as binary variables, with male coded as 1 and female as 0, and rural residence coded as 1 and urban residence as 0. Separate age-category coefficients were included to capture potential non-linear associations between age and diagnostic outcomes, with young adults (<40 years) serving as the reference group. Predefined interaction terms between sex, age group, and area of residence were included based on biological plausibility and previous epidemiological evidence suggesting that access to dental care, healthcare-seeking behavior, and disease progression may differ across demographic and residential subgroups [5,10,12,14,15,16,17,18,19,20,21]. All interaction terms were specified *a priori* before statistical modeling.

Multicollinearity was assessed using the Variance Inflation Factor (VIF), with a VIF value below 5 considered acceptable. Because all logistic models used the same structure of independent predictors, VIF analysis was performed only once for the predictor matrix, with the values being applicable to all models. The Hosmer–Lemeshow goodness-of-fit test was employed to evaluate the correspondence between observed and predicted frequencies. Model explanatory power was estimated using McFadden’s Pseudo-R^2^. The same procedure—individual logistic regression models—was used for each tooth-type category. The statistical analysis was performed using Stata v19.5 (StataCorp, College Station, TX, USA), with statistical significance defined as a *p*-value less than 0.05.

## 3. Results

### 3.1. Population Characteristics

The study population included 872 men (52.85%) and 778 women (47.15%), with fewer rural (*n* = 730, 44.25%) than urban residents (*n* = 920, 55.75%). Most patients were young adults (*n* = 1226, 74.31%), while middle-aged adults (*n* = 320, 19.39%) and older adults (*n* = 104, 6.30%) represented only one-quarter of the entire cohort. The diagnosis of other dental conditions was the most common primary diagnosis (*n* = 559, 33.87%), followed by endodontic conditions (*n* = 449, 27.21%) and periodontal diseases (*n* = 417, 25.27%). In contrast, acute infections (*n* = 129, 7.81%) and carious lesions (*n* = 96, 5.82%) were less frequent. The distribution of specific conditions within each category is given in Table 2. Within the K08.x category, loss of teeth predominated (*n* = 215, 41.75%), followed by retained dental roots (*n* = 145, 28.16%), other specified disorders (*n* = 88, 17.09%), unspecified disorders (*n* = 42, 8.16%), alveolar ridge atrophy (*n* = 14, 2.72%), and exfoliation of teeth (*n* = 11, 2.14%). The distribution of variables across the analyzed cases is given in Supplementary Table S1. No caries cases were identified among older adults based on the available coding data.

### 3.2. Association Between Sociodemographic Factors and Dental Conditions

The results of the logistic regression, with dental condition as the outcome, are presented in Table 3. Regression model evaluation confirmed the adequacy and stability of all analyses. We found an acceptable predictor overlap, with VIF values below 4 for all variables (VIF ≤ 3.11). All models demonstrated event-per-variable (EPV) ratios above the standard threshold of 10 events per predictor (EPV ≥ 14.3 cases), while Hosmer–Lemeshow tests indicated satisfactory goodness-of-fit (*p* ≥ 0.609; McFadden’s Pseudo-R^2^ ≤ 0.187).

Middle-aged patients showed lower odds of dental caries and endodontic conditions than young individuals. Older adults were less likely to present with endodontic diseases. In addition, rural inhabitants showed a higher likelihood of endodontic pathologies versus urban residents. This association differed significantly by sex, with rural men revealing twofold higher odds of this outcome compared to the reference group (urban women). Both older and middle-aged individuals showed nearly twofold increased likelihood of having periodontal diseases. Moreover, rural residents were more likely to present with this diagnosis. No other significant associations or interactions were detected.

Female sex, urban residence, and age < 40 years were used as reference categories throughout all models. Predictor labels are presented as comparisons against these reference groups. Interaction terms represent combined effects beyond the corresponding main effects. Bold values denote statistically significant associations.

### 3.3. Association Between Sociodemographic Factors and Affected Tooth Types

The distribution of affected tooth types across sex, area of residence, and age strata is given in Table 4. Molars were the most frequently affected tooth type in both sexes, particularly among females, whereas anterior teeth were more commonly involved in males. Rural and urban populations showed similar molar and premolar distributions, although anterior tooth involvement was more frequent in rural residents. Across age groups, the proportion of cases involving molars tended to be lower in older individuals, whereas the proportion involving anterior teeth tended to be higher.

The results of the logistic model, using affected tooth type as the outcome, are given in Table 5. Each model satisfied the conventional adequacy criterion of more than 10 events (EPV ≥ 23.3). Nonsignificant goodness-of-fit results were observed across all tooth types (*p* ≥ 0.531; McFadden’s Pseudo-R^2^ ≤ 0.131), supporting adequate model calibration. Sex was the only variable independently associated with affected tooth type. However, these relationships were inverse: male patients were more likely to have affected anterior teeth but less likely to have affected molars. Although age-related differences in tooth-type involvement were observed in the unadjusted analyses, these associations were no longer significant after multivariable adjustment, suggesting that the observed variation was largely explained by other factors included in the model. No significant associations were identified for the remaining predictors or interaction terms.

## 4. Discussion

This study integrates demographic (age, sex) and residential (rural–urban) factors to understand their effect on dental disease distribution in a relatively large cohort and uses real-world clinical data derived from a major public hospital. Using a single multivariate framework, we identified both age and area of residence as independent predictors of periodontal disease and endodontic conditions, as well as a significant interaction between sex and area of residence in endodontic conditions. Our results also point toward differential patterns of dental involvement based on sex, with anterior tooth involvement more common in men and molar involvement more prevalent in women. With a slight bias toward males over females, the sex ratio in our cohort is in line with oral health data from the National Hospital Ambulatory Medical Care Survey (United States) or comparable tertiary care hospitals, such as Mainz University Hospital (Germany) and Kaohsiung Medical University Hospital (Taiwan) [37,38,39,40]. The observation that most cases were younger than 40 years is consistent with the dental emergency literature; this shows clear peaks in the use of emergency dental services in young adults (e.g., 18–44 years in the US; 20–44 years in Canada; 20–40 years in Germany and Romania) [16,17,23,40,41,42,43]. Our cohort included fewer rural residents; these populations are likely to exhibit higher risk of late presentation to the emergency department due to less frequent dental visits and lower access to preventive care [44].

Although carious lesions were recorded as the primary diagnosis in only 96 patients (5.82%), this figure should not be interpreted as the overall burden of caries-related conditions in our cohort. When caries and its likely downstream endodontic sequelae are considered together, 545 patients (33.03%) presented with conditions potentially related to caries progression. The primary-diagnosis approach used in this study may therefore underestimate caries as an etiological contributor while still capturing the principal clinical condition responsible for emergency presentation.

The low prevalence of dental caries may originate from the frequent misclassification as “pulp/periapical disease” (K04) or “other dental disorders” (K08) in emergency settings, the limitations of administrative coding (coder–dentist discordance), or the clinical focus on symptomatic presentation [45]. We also noted the high prevalence of “other diagnoses” (K08.x) and periodontitis (K05.3)—together, these categories accounted for more than half of all clinical cases. This finding may reflect a clinical environment predominantly focused on managing the consequences of untreated dental decay and advanced periodontal destruction. Furthermore, the relatively low incidence of carious lesions, but high rates of pulpitis and periapical abscesses may stem from diagnostic displacement, with patients entering the healthcare system when their condition has progressed to a state of acute pain or functional impairment. Since the former diagnosis reflects edentulism and the chronic consequences of extractions, its high prevalence may indicate the preponderance of surgical interventions over restorative care in this cohort. In emergency settings, K08 codes are among the most frequently listed primary diagnoses, often surpassing caries in treat-and-release visits. Specifically, loss of teeth and similar disorders account for over one-third of dental-related treat-and-release emergency dental visits in North American data [41]. The marked disparity between the prevalence of gingivitis and periodontitis—the latter being more than tenfold higher—may be indicative of a clinical setting that primarily addresses advanced pathological stages with irreversible tissue loss.

Consistent with dental literature data [1,6,33], middle-aged adults showed a lower likelihood of caries compared to young adults. However, this effect in older adults remains difficult to quantify reliably due to small sample size. The low frequency of early-stage conditions in these patients may derive from the cumulative effect of disease progression, delayed healthcare-seeking behavior, increased tooth loss, and ICD-10 coding practices prioritizing advanced diagnoses [6]. We also observed reduced odds of endodontic conditions, but higher odds of periodontal diseases in middle-aged and older individuals versus young individuals. The lower odds of endodontic conditions in older adults may originate from the age-related structural changes in the pulp–dentin complex, including reduced pulp chamber volume and dentinal sclerosis; these changes may limit the progression of acute pulpal inflammation [46,47,48].

Rural living was associated with a one-and-a-half-fold increase in the odds of endodontic pathology or endodontic complications. This magnitude may seem larger than in most studies of more broadly defined emergency dental visits. For example, Akinlotan et al. found that the effect of rurality is typically more modest for emergency visits related to non-traumatic dental conditions [14]. However, our results are plausible if the outcome is strictly endodontic and analysis is conditional on the patient having already reached the emergency department. The aforementioned finding is consistent with the known access barriers in rural populations [44,49,50]. This may contribute to the progression of untreated carious lesions to pulpal and periapical involvement, increasing the likelihood of endodontic treatment, endodontic failure, or periapical complications [7]. For Romania, the structural context already described above makes the association even more credible.

The significant interaction between sex and area of residence in endodontic conditions suggests that rural disadvantage may affect men and women differently. Data from the Centers for Disease Control and Prevention (United States) showed that the most disadvantaged group—in terms of dental service utilization—is precisely that of rural men: only 53.5% reported a dental visit in the last 12 months, compared to 70.5% of urban women, 62.7% of urban men, and 61.4% of rural women [44]. This gradient is compatible with the distribution of patients by sex and area of residence in our cohort. Literature data support the existence of different behavioral patterns, with women more proactive in seeking advice or initial care for pain and men postponing presentation until surgical or traumatic emergencies develop [14,51]. These differences in service utilization can translate into later diagnosis, longer untreated caries, progression to pulpitis/apical periodontitis, treatment in more advanced stages, and higher risk of complications or failure.

Clinical data demonstrate a substantial increase in the prevalence of periodontal diseases after the age of 40 years, with the steepest rise from the fourth to the fifth decade of life [2,4,5,15,52]. This trend is in line with our findings. Beyond the age-related biological susceptibility, the area of residence emerged as an independent predictor of periodontal burden. The higher likelihood of rural residents being diagnosed with these conditions is congruent with epidemiological surveys across the entire spectrum of income-level nations [3,5,12,52,53,54]. This pattern may reflect differences in access to dental care, healthcare utilization, or other unmeasured factors. The predominance of periodontitis over gingivitis in the study population may further suggest that many patients presented when the disease had already progressed to a more advanced stage.

Although molars accounted for the largest proportion of affected teeth across all age groups, a non-significant trend toward lower molar involvement and greater anterior tooth involvement was observed among older individuals. Potential explanations include longer lifetime exposure to dental disease, prior extractions, and age-related periodontal changes [10,55], although these interpretations remain speculative. Interestingly, sex was the only significant predictor of affected tooth type, but this association was limited to anterior teeth and molars. The present data do not permit conclusions regarding the mechanisms underlying these differences, but existing literature hints at several potential contributing factors. Most studies report a female excess for molar involvement (especially in the first molars), possibly related to earlier tooth eruption in girls, hormonal and salivary differences, or sex-related eating/behavioral roles [26,27,56,57,58]. On the other hand, male sex appears to be associated with greater involvement of anterior teeth (particularly for untreated caries) [29,59,60]. Men more frequently engage in specific cariogenic behaviors, such as consumption of sugary beverages and smoking, while also being more commonly affected by class IV mesial caries (often due to trauma/neglect) [14,60]. Given the modest effect sizes observed in the present study, however, these findings should be interpreted cautiously and confirmed in future investigations.

While this study provides valuable insights, our findings should be interpreted in light of certain methodological constraints. Given the single-center retrospective design of our study, the present results may not be applicable to patients under different circumstances (hospitals, countries, demographics) and may be subject to multiple sources of systematic bias, incomplete data, and unmeasured confounds. Although possible ICD-10 coding bias may distort proportions and odds ratios, classification into broad categories (caries vs. endodontic vs. periodontal, etc.) reduces the impact of fine coding errors and facilitates population-level analyses [45]. On the other hand, the absence of socioeconomic and behavioral information may prevent adjustment for factors influencing dental disease patterns (e.g., income, education, smoking, hygiene). Although area of residence may serve as a proxy for healthcare access and resource availability in settings characterized by unequal distribution of dental care providers [18], residual confounding from unmeasured socioeconomic and behavioral factors cannot be excluded. Therefore, the observed associations with rural residence should be interpreted as associations rather than evidence of a direct causal effect.

Another important drawback involves the hierarchical diagnostic classification; this approach may have underestimated the contribution of carious lesions to the overall burden of emergency dental disease. Since in our study no caries cases were observed among patients aged ≥60 years, the association between older age and dental caries could not be reliably estimated and should be interpreted with caution. In addition, the modest McFadden’s Pseudo-R^2^ values indicate that the included predictors explained only part of the variability in dental pathology outcomes, suggesting that additional clinical, behavioral, and environmental factors may contribute to the observed patterns. Furthermore, information on dental status indicators, such as number of teeth and edentulism, was unavailable. The World Health Organization (WHO) includes edentulism as a separate indicator, and the fact that edentulism increases with age is sufficiently well known that the mechanism remains plausible [60]. Even if edentulism can account for part of these findings, the increase in periodontal disease with age remains informative and relevant for service planning [5]. Finally, all analyses were specified *a priori* based on biological and epidemiological plausibility, with multiple statistical comparisons being performed. Therefore, the possibility of Type I error, particularly for associations with borderline statistical significance, cannot be entirely excluded, and such findings should be interpreted with caution and confirmed in future studies.

## 5. Conclusions

This study suggests that patterns of emergency dental presentations are associated with age, area of residence, and sex within this tertiary-care cohort. Periodontal diseases were more commonly observed among middle-aged and older adults, whereas endodontic conditions were more frequent among younger individuals and rural residents. The observed interaction between sex and area of residence support that rural men may represent a subgroup with a higher burden of endodontic pathology. In addition, male patients were more likely to present with anterior tooth involvement and less likely to present with molar involvement. Overall, the predominance of advanced dental conditions and the relatively low proportion of carious lesions recorded as primary diagnoses may be consistent with delayed utilization of preventive and restorative dental services. These findings may help inform future oral health initiatives and support further investigation of demographic and rural–urban disparities in dental emergency care.

## Figures and Tables

**Table 1:** Inclusion and exclusion criteria.

Inclusion Criteria	Exclusion Criteria
Aged 18 years or older; Availability of complete and analyzable clinical records; Presence of at least one clinically established dental diagnosis; First recorded emergency dental visit during the study period; Treatment performed between 1 January and 28 August 2025; Availability of documented consent for anonymized data use.	Incomplete or missing essential data; Ambiguous, inconsistent, or non-standardized diagnostic entries; Duplicate record or repeated visits; Cases lacking valid informed consent for the use of anonymized clinical data; Patients presenting exclusively for administrative purposes without clinical evaluation; Implausible values suggestive of data entry errors.

**Table 2:** Classification of dental conditions and corresponding ICD-10 codes.

Category	Component Diagnosis	ICD-10	*n* (%)
Carious lesions	Dental caries	K02.x	96 (5.82%)
Endodontic conditions	Pulpitis	K04.0	322 (19.52%)
	Periapical abscess	K04.7	127 (7.70%)
Periodontal diseases	Periodontitis	K05.3	379 (22.97%)
	Gingivitis	K05.0–1	38 (2.30%)
Acute infections	Cellulitis (oral tissues)	K12.2	127 (7.70%)
	Osteitis (osteomyelitis)	K10.2	2 (0.11%)
Other dental pathologies	Trauma	S02.5	44 (2.67%)
	Other diagnoses	K08.x	515 (31.21%)

Data in the fourth column are shown as counts with percentages (in parentheses).

**Table 3:** Effect of age, sex, area of residence, and their interactions on dental conditions.

Outcome	Predictor	β	SE	OR (95% CI)	Wald (Z)	*p*
Carious lesions	Sex: M vs. F	0.59	0.48	1.81 (0.71–4.60)	1.24	0.214
	Age: 40–59 vs. <40	−1.56	0.62	0.21 (0.06–0.68)	−2.52	0.009 **
	Area of residence: Rural vs. Urban	0.63	0.52	1.88 (0.68–5.19)	1.22	0.220
	Sex: M vs. F× Age: 40–59 vs. <40	0.18	1.28	1.20 (0.10–14.82)	0.14	0.887
	Sex: M vs. F× Area of residence: Rural vs. Urban	−0.25	0.63	0.78 (0.23–2.66)	−0.40	0.692
	Area of residence: Rural vs. Urban× Age: 40–59 vs. <40	−0.68	1.27	0.51 (0.04–6.13)	−0.54	0.592
Endodontic conditions	Sex: M vs. F	−0.35	0.23	0.70 (0.45–1.10)	−1.53	0.126
	Age: 40–59 vs. <40	−0.87	0.38	0.42 (0.20–0.88)	−2.29	0.011 *
	Age: ≥60 vs. <40	−0.54	0.21	0.58 (0.39–0.88)	−2.57	0.021 *
	Area of residence: Rural vs. Urban	0.45	0.23	1.57 (1.00–2.46)	1.98	0.048 *
	Sex: M vs. F× Age: 40–59 vs. <40	0.51	0.43	1.66 (0.71–3.88)	1.18	0.239
	Sex: M vs. F× Age: ≥60 vs. <40	1.12	0.88	3.07 (0.54–17.31)	1.27	0.203
	Sex: M vs. F× Area of residence: Rural vs. Urban	0.71	0.33	2.03 (1.06–3.89)	2.12	0.033 *
	Area of residence: Rural vs. Urban× Age: 40–59 vs. <40	−0.49	0.46	0.61 (0.25–1.50)	−1.08	0.282
	Area of residence: Rural vs. Urban× Age: ≥60 vs. <40	−0.11	0.81	0.89 (0.18–4.42)	−0.14	0.891
Periodontal diseases	Sex: M vs. F	0.02	0.26	1.02 (0.62–1.69)	0.09	0.931
	Age: 40–59 vs. <40	0.7	0.19	2.02 (1.40–2.91)	3.68	<0.001 ***
	Age: ≥60 vs. <40	0.65	0.29	1.92 (1.08–3.42)	2.24	0.026 *
	Area of residence: Rural vs. Urban	0.57	0.28	1.76 (1.02–3.05)	2.01	0.045 *
	Sex: M vs. F× Age: 40–59 vs. <40	−0.28	0.38	0.76 (0.36–1.59)	−0.74	0.460
	Sex: M vs. F× Age: ≥60 vs. <40	0.08	0.61	1.08 (0.33–3.54)	0.13	0.900
	Sex: M vs. F× Area of residence: Rural vs. Urban	−0.35	0.33	0.70 (0.37–1.34)	−1.07	0.282
	Area of residence: Rural vs. Urban× Age: 40–59 vs. <40	−0.21	0.38	0.81 (0.38–1.73)	−0.54	0.591
	Area of residence: Rural vs. Urban× Age: ≥60 vs. <40	−0.79	0.65	0.45 (0.13–1.61)	−1.22	0.221
Acute infections	Sex: M vs. F	−0.36	0.38	0.70 (0.33–1.48)	−0.94	0.348
	Age: 40–59 vs. <40	−0.14	0.51	0.87 (0.32–2.39)	−0.26	0.791
	Age: ≥ 60 vs. <40	−0.63	1.07	0.53 (0.07–4.36)	−0.58	0.559
	Area of residence: Rural vs. Urban	−0.29	0.43	0.75 (0.32–1.73)	−0.68	0.497
	Sex: M vs. F× Age: 40–59 vs. <40	0.72	0.64	2.06 (0.58–7.23)	1.12	0.261
	Sex: M vs. F× Age: ≥60 vs. <40	1.38	1.21	3.98 (0.37–42.51)	1.14	0.253
	Sex: M vs. F× Area of residence: Rural vs. Urban	0.33	0.55	1.39 (0.47–4.11)	0.59	0.553
	Area of residence: Rural vs. Urban× Age: 40–59 vs. <40	−0.48	0.69	0.62 (0.16–2.39)	−0.69	0.487
	Area of residence: Rural vs. Urban× Age: ≥60 vs. <40	−0.75	1.22	0.47 (0.04–5.16)	−0.61	0.538
Other dental pathologies	Sex: M vs. F	0.14	0.21	1.15 (0.76–1.74)	0.68	0.498
	Age: 40–59 vs. <40	−0.19	0.29	0.83 (0.47–1.46)	−0.66	0.508
	Age: ≥60 vs. <40	0.4	0.48	1.49 (0.58–3.80)	0.83	0.405
	Area of residence: Rural vs. Urban	−0.1	0.24	0.91 (0.57–1.44)	−0.41	0.678
	Sex: M vs. F× Age: 40–59 vs. <40	0.06	0.35	1.06 (0.53–2.11)	0.17	0.868
	Sex: M vs. F× Age: ≥60 vs. <40	−0.51	0.57	0.60 (0.19–1.84)	−0.89	0.371
	Sex: M vs. F× Area of residence: Rural vs. Urban	−0.29	0.29	0.75 (0.42–1.32)	−1.000	0.315
	Area of residence: Rural vs. Urban× Age: 40–59 vs. <40	0.52	0.36	1.68 (0.83–3.40)	1.45	0.148
	Area of residence: Rural vs. Urban× Age: ≥60 vs. <40	0.78	0.6	2.18 (0.68–7.03)	1.31	0.190

β, beta coefficient; SE, standard error; OR, adjusted odds ratio with 95% confidence interval; Wald(Z), z test value; *p*, *p* value; F, female sex; M, male sex; <40, under 40 years old (young adults); 40–59, aged 40–59 years (middle-aged adults); ≥60, 60 years and older (older adults). Female sex, urban residence, and age <40 years were used as reference categories throughout all models. Predictor labels are presented as comparisons against these reference groups. Interaction terms represent combined effects beyond the corresponding main effects. Bold values denote statistically significant associations. The ≥60 age category was omitted from the caries model due to complete separation (zero events). Bold values marked with asterisks (*) identify significant predictors of specific dental pathologies (Wald test, ***—*p* ≤ 0.001; **—*p* ≤ 0.01; and *—*p* ≤ 0.05).

**Table 4:** Distribution of different tooth types across sex, area of residence, and age strata.

Tooth Type	Sex		Area of Residence		Age		
	Male	Female	Rural	Urban	<40	40–59	≥60
Anterior	139 (15.94%)	71 (9.13%)	92 (12.60%)	58 (6.30%)	87 (7.10%)	88 (27.50%)	36 (34.62%)
Molar	477 (54.70%)	491 (63.11%)	387 (53.01%)	550 (59.78%)	775 (63.21%)	150 (46.88%)	41 (39.42%)
Premolar	256 (29.36%)	216 (27.76%)	251 (34.38%)	312 (33.91%)	364 (29.69%)	82 (25.63%)	27 (25.96%)

<40, under 40 years old (young adults); 40–59, aged 40–59 years (middle-aged adults); ≥60, 60 years and older (older adults). Data are shown as counts with percentages (in parentheses).

**Table 5:** Effect of sex, age, area of residence, and their interactions on affected tooth type.

Outcome	Predictor	β	SE	OR (95% CI)	Wald (Z)	*p*
Anterior teeth	Sex: M vs. F	0.48	0.24	1.62 (1.01–2.60)	2.00	0.045 *
	Age: 40–59 vs. <40	−0.31	0.28	0.73 (0.42–1.28)	−1.11	0.268
	Area of residence: Rural vs. Urban	−0.52	0.46	0.59 (0.24–1.45)	−1.13	0.257
	Sex: M vs. F× Age: 40–59 vs. <40	−0.10	0.27	0.90 (0.53–1.53)	−0.37	0.710
	Sex: M vs. F× Age: ≥60 vs. <40	0.21	0.41	1.23 (0.55–2.75)	0.51	0.608
	Sex: M vs. F× Area of residence: Rural vs. Urban	0.62	0.71	1.86 (0.46–7.43)	0.87	0.383
	Area of residence: Rural vs. Urban× Age: 40–59 vs. <40	0.28	0.34	1.32 (0.68–2.57)	0.82	0.412
Molar	Sex: M vs. F	−0.37	0.18	0.69 (0.47–0.99)	−2.03	0.042 *
	Age: 40–59 vs. <40	0.18	0.23	1.20 (0.76–1.88)	0.78	0.436
	Age: ≥60 vs. <40	0.34	0.38	1.40 (0.67–2.93)	0.89	0.373
	Area of residence: Rural vs. Urban	−0.06	0.22	0.94 (0.61–1.46)	−0.27	0.786
	Sex: M vs. F× Age: 40–59 vs. <40	−0.29	0.32	0.75 (0.40–1.42)	−0.91	0.363
	Sex: M vs. F× Age: ≥60 vs. <40	−0.81	0.59	0.44 (0.14–1.37)	−1.37	0.170
	Sex: M vs. F× Area of residence: Rural vs. Urban	−0.22	0.28	0.80 (0.46–1.41)	−0.79	0.429
	Area of residence: Rural vs. Urban× Age: 40–59 vs. <40	0.14	0.33	1.15 (0.60–2.20)	0.42	0.672
	Area of residence: Rural vs. Urban× Age: ≥60 vs. <40	0.52	0.60	1.68 (0.52–5.41)	0.87	0.384
Premolar	Sex: M vs. F	0.10	0.22	1.10 (0.72–1.68)	0.45	0.652
	Age: 40–59 vs. <40	0.08	0.25	1.08 (0.66–1.76)	0.32	0.750
	Age: ≥60 vs. <40	0.18	0.40	1.20 (0.55–2.61)	0.45	0.653
	Area of residence: Rural vs. Urban	0.44	0.24	1.55 (0.97–2.47)	1.83	0.067
	Sex: M vs. F× Age: 40–59 vs. <40	0.06	0.34	1.06 (0.54–2.07)	0.18	0.857
	Sex: M vs. F× Age: ≥60 vs. <40	−0.49	0.58	0.61 (0.20–1.86)	−0.84	0.401
	Sex: M vs. F× Area of residence: Rural vs. Urban	−0.31	0.30	0.73 (0.41–1.31)	−1.03	0.303
	Area of residence: Rural vs. Urban× Age: 40–59 vs. <40	0.51	0.36	1.67 (0.82–3.40)	1.42	0.155
	Area of residence: Rural vs. Urban× Age: ≥60 vs. <40	0.79	0.61	2.20 (0.66–7.29)	1.30	0.193

β, beta coefficient; SE, standard error; OR, adjusted odds ratio with 95% confidence interval; Wald(Z), z test value; *p*, *p* value; F, female sex; M, male sex; <40, under 40 years old (young adults); 40–59, aged 40–59 years (middle-aged adults); ≥60, 60 years and older (older adults). Female sex, urban residence, and age <40 years were used as reference categories throughout all models. Predictor labels are presented as comparisons against these reference groups. Interaction terms represent combined effects beyond the corresponding main effects. Bold values denote statistically significant associations. Bold values marked with asterisks (*) identify significant predictors of specific dental pathologies (Wald test, ***—*p* ≤ 0.001; **—*p* ≤ 0.01; and *—*p* ≤ 0.05).

## Data Availability

The data presented in this study are available on request from the corresponding author. The data are not publicly available due to privacy.

## Supplementary Materials

The following supporting information can be downloaded at: https://www.mdpi.com/article/10.3390/healthcare14131914/s1, Supplementary Table S1: The distribution of the analyzed variables across the study entries.

## Abbreviations

The following abbreviations are used in this manuscript:

OR	Odds ratio
CI	95% confidence interval
ICD-10	International Classification of Diseases, 10th Revision
β	Beta coefficient
SE	Standard error
Wald (Z)	Wald statistic (Z-test value)
EU	European Union
WHO	World Health Organization
