# Supplementary material for: Rural–Urban and Demographic Inequalities in Dental Pathology and Diagnostic Patterns in Romanian Emergency Care: A Retrospective Tertiary Clinical Cohort Study
Source: Healthcare (Basel). 2026 Jul 1;14(13):1914. doi: 10.3390/healthcare14131914 (PMC13361455; doi:10.3390/healthcare14131914)
Supplement: Supplementary file 1 [file healthcare-14-01914-s001.zip › healthcare-4366380-supplementary.pdf]

Table S1. The distribution of the analyzed variables across the study entries.

Sex	Age group	Area of residence	Total	Carious lesions	Endodontic conditions	Periodontal diseases	Acute infections	Other dental pathologies
F	<40	Rural	314	25 (7.96%)	84 (26.75%)	87 (27.71%)	26 (8.28%)	92 (29.30%)
F	<40	Urban	268	11 (4.10%)	92 (34.33%)	51 (19.03%)	22 (8.21%)	92 (34.33%)
F	40–59	Rural	57	0 (0.00%)	4 (7.02%)	24 (42.11%)	0 (0.00%)	29 (50.88%)
F	40–59	Urban	102	2 (1.96%)	24 (23.53%)	39 (38.24%)	11 (10.78%)	26 (25.49%)
F	≥60	Rural	8	0 (0.00%)	0 (0.00%)	2 (25.00%)	0 (0.00%)	6 (75.00%)
F	≥60	Urban	29	0 (0.00%)	4 (13.79%)	11 (37.93%)	2 (6.90%)	12 (41.38%)
M	<40	Rural	270	27 (10.00%)	86 (31.85%)	57 (21.11%)	17 (6.30%)	83 (30.74%)
M	<40	Urban	374	27 (7.22%)	105 (28.07%)	75 (20.05%)	27 (7.22%)	140 (37.44%)
M	40–59	Rural	60	2 (3.33%)	14 (23.33%)	18 (30.00%)	7 (11.67%)	19 (31.67%)
M	40–59	Urban	101	2 (1.98%)	23 (22.77%)	31 (30.69%)	9 (8.91%)	36 (35.65%)
M	≥60	Rural	21	0 (0.00%)	5 (23.81%)	5 (23.81%)	2 (9.52%)	9 (42.86%)
M	≥60	Urban	46	0 (0.00%)	8 (17.39%)	17 (36.96%)	6 (13.04%)	15 (32.61%)

F, female sex; M, male sex; <40, under 40 years old; 40-59, aged 40-59 years; ≥60, 60 years and older. Data are shown as counts with percentages (in parentheses).
